# Doctors’ attitudes to, beliefs about, and experiences of the regulation of professional competence: a scoping review protocol

**DOI:** 10.1186/s13643-019-1132-3

**Published:** 2019-08-22

**Authors:** Anél Wiese, Emer Galvin, Charlotte Merrett, Irina Korotchikova, Dubhfeasa Slattery, Lucia Prihodova, Hilary Hoey, Ann O’Shaughnessy, Jantze Cotter, Janet O’Farrell, Mary Horgan, Deirdre Bennett

**Affiliations:** 1 Medical Education Unit, School of Medicine, University College Cork, Cork, Ireland; 2 Royal College of Surgeons in Ireland, Dublin, Ireland; 3 Royal College of Physicians of Ireland, Dublin, Ireland; 4 Medical Council, Dublin, Ireland

**Keywords:** Regulation of professional competence, Scoping review, Revalidation, Recertification, Maintenance of certification

## Abstract

**Background:**

Historically, individual doctors were responsible for maintaining their own professional competence. More recently, changing patient expectations, debate about the appropriateness of professional self-regulation, and high-profile cases of malpractice have led to a move towards formal regulation of professional competence (RPC). Such programmes require doctors to demonstrate that they are fit to practice, through a variety of means. Participation in RPC is now part of many doctors’ professional lives, yet it remains a highly contested area. Cost, limited evidence of impact, and lack of relevance to practice are amongst the criticisms cited. Doctors’ attitudes towards RPC, their beliefs about its objectives and effectiveness, and their experiences of trying to meet its requirements can impact engagement with the process. We aim to conduct a scoping review to map the empirical literature in this area, to summarise the key findings, and to identify gaps for future research.

**Methods:**

We will conduct our review following the six phases outlined by Arksey and O’Malley, and Levac. We will search seven electronic databases: Academic Search Complete, Business Source Complete, CINAHL, PsycINFO, PubMed, Social Sciences Full Text, and SocINDEX for relevant publications, and the websites of medical regulatory and educational organisations for documents. We will undertake backward and forward citation tracking of selected studies and will consult with international experts regarding key publications. Two researchers will independently screen papers for inclusion and extract data using a piloted data extraction tool. Data will be collated to provide a descriptive summary of the literature. A thematic analysis of the key findings will be presented as a narrative summary of the literature.

**Discussion:**

We believe that this review will be of value to those tasked with the design and implementation of RPC programmes, helping them to maximise doctors’ commitment and engagement, and to researchers, pointing to areas that would benefit from further enquiry. This research is timely; internationally existing programmes are evolving, new programmes are being initiated, and many jurisdictions do not yet have programmes in place. There is an opportunity for learning across different programmes and from the experiences of established programmes. Our review will support that learning.

**Systematic review registration:**

PROSPERO does not register scoping reviews.

**Electronic supplementary material:**

The online version of this article (10.1186/s13643-019-1132-3) contains supplementary material, which is available to authorized users.

## Background

The medical profession and its members are collectively and individually accountable for the delivery of safe patient care. Historically, individual doctors, once qualified, were responsible for maintaining their own career-long professional competence without external review. In recent decades, increased patient expectations about the quality of care, debate about the appropriateness of professional self-regulation [1, 2], and high-profile cases of malpractice [3] have led to the international implementation of programmes aimed at validating the continuing competence of doctors [4, 5]. These programmes require doctors to regularly demonstrate that they are up-to-date and fit to practice [6].

To date, one peer-reviewed scoping review has been published on this topic which described the requirements of programmes for validating doctors’ competence and fitness to practice in four countries and offered a summary of the terminology used [7]. A wide variety of terms are used to describe these programmes including ‘revalidation’, ‘recertification’, ‘relicensing’, ‘maintenance of certification’, and ‘maintenance of licensure’ [6, 7]. Their goal is to ensure high-quality and safe medical care by prompting doctors to maintain their clinical knowledge, skills, and professional behaviours through lifelong learning, thus encouraging compliance with professional standards, and enhancing the continuing professional development (CPD) of doctors in their chosen specialty [6, 8–10]. We use the term ‘regulation of professional competence’ (RPC) to refer to programmes implemented with these objectives in mind.

RPC programme requirements vary from country to country but, typically, involve a combination of learning and assessment elements such as participation in knowledge self-assessments, examinations, quality improvement projects or practice audits, appraisal, peer and patient feedback, and CPD [6, 10, 11]. The debate continues as to whether RPC has been effective in achieving these outcomes [12–14].

The development and implementation of RPC programmes pose several challenges. Cost of participating in RPC and limited evidence that RPC improves patient outcomes have undermined doctors’ commitment to RPC programmes [15]. RPC has been criticised as being a ‘tick-box’ exercise and an unnecessary bureaucratic burden for doctors and employers [6]. Doctors have expressed concerns about RPC programmes’ relevance [16], and ensuring that RPC applies to doctors’ daily practice remains a challenge [14, 15].

Regulation of professional competence is, and will continue to be, an essential part of many doctors’ careers, yet it remains a highly contested area. Doctors’ attitudes towards RPC, their beliefs about its objectives and effectiveness, and their experiences of trying to meet its requirements can act as barriers to meaningful engagement with the process. Preliminary searches have indicated that a significant body of work on doctors’ perceptions and experiences of RPC exists; however, there is no published synthesis of the literature on this specific topic. Therefore, we aim to conduct a scoping review to map the empirical literature in this area, to summarise the key findings, and to identify gaps for future research.

Scoping reviews aim to map the extent of evidence [17]; therefore, we have developed research questions that are sufficiently broad to ensure a breadth of coverage of the topic under review. The scoping review will aim to address the following research questions:

1. What empirical research has been published about doctors’ attitudes to, beliefs about, and experiences of RPC programmes?
2. What are doctors’ attitudes to, beliefs about, and experiences of RPC programmes?
3. What are the gaps in the literature related to doctors’ experiences, attitudes, and beliefs about existing RPC programmes?

## Methods

Scoping review is a method used to comprehensively map the literature available on a topic and involves systematically identifying the key concepts, underpinning theories, sources of evidence, and gaps in the research [17, 18]. This protocol was designed according to the PRISMA Extension for Scoping Reviews (PRISMA-ScR) reporting guidelines [19] (see checklist in Additional file 1). The scoping review will be conducted following the framework developed by Arksey and O’Malley [20] and advanced by Levac and colleagues [17] which comprises six steps: (1) identifying the research question; (2) identifying relevant studies; (3) study selection; (4) charting the data; (5) collating, summarising, and reporting the results; and (6) consultation.

### Eligibility criteria

Publications will be considered eligible if they meet the following selection criteria.

Inclusion criteria:

- Published in the English language
- Literature relating to doctors’ attitudes to, beliefs about, and experiences of RPC as a whole, and/or individual elements of RPC programmes
- Literature relating to RPC programmes in the UK, Ireland, Canada, USA, Australia, and New Zealand
- Peer-reviewed research including empirical papers and commentaries or letters containing empirical data
- Government or organisational reports and consultation documents containing empirical data relevant to the focus of the review

Exclusion criteria:

- Literature on RPC relating to healthcare professionals other than doctors (e.g. nursing)
- Non-peer-reviewed commentaries, reviews, and letters
- Grey literature other than the reports and consultation documents referred to in the inclusion criteria

Following preliminary exploratory searches, we chose to limit our search to empirical data to countries that have a system of regulation of professional competence in place or were about to launch such a system. The rationale for this decision was our focus on doctors’ attitudes and beliefs grounded in experiences of actual programmes of RPC rather more abstracted attitudes towards the notion of regulation of professional competence.

### Information sources

Comprehensiveness and breadth are essential in the search for relevant studies [17]; therefore, the research team will use a three-step approach to identify relevant publications. The first step will be to search seven electronic databases: Academic Search Complete, Business Source Complete, CINAHL, PsycINFO, PubMed, Social Sciences Full Text, and SocINDEX. Search terms will include Medic*, Revalidation, Appraisal, Maintenance and “Medical Licensure”, Maintenance and Certification, “Medical Registration”, Maintenance and “Professional Competence”. Search terms will be adapted to the requirements of each database. The second step will entail searching the websites of selected countries’ medical regulatory and educational organisations for relevant research reports and policy documents. In an iterative process, following completion of the selection of sources of evidence (below), the third step will involve backward and forward citation tracking of selected studies. Papers identified during these searches will be imported and managed using Endnote X8 [21], and duplicates removed.

#### Consultation

We will conduct interviews with international stakeholders to gain additional insights into RPC in their country of practice (i.e. USA, Canada, Australia, New Zealand, UK, and Ireland). The consultations will be based on the preliminary findings of the review. The interview questions will include background information on national mechanisms for RPC, as well as doctors’ engagement with and commitment to the process, management of non-compliance, and innovations designed to enhance engagement. Participants will also be asked to identify key policy documents and other relevant sources of empirical data to include in the review. Interviewees will provide insights, expertise, and perspective on the initial review results. Interviews will be professionally transcribed and analysed thematically, and the information provided synthesised with that found in the scoping review. We have received ethical approval for the consultation interviews from the Social Research Ethics Committee, University College Cork.

### Search

The search strategy was developed by the core research team in collaboration with an information specialist and the wider project team. Table 1 outlines the full electronic search strategy that will be used in PubMed.

**Table 1:** Electronic search strategy (PubMed)

Database: PubMed		
Search strategy	Query	Search details
#1	(medic*) AND revalidation Filters: English	(medic[All Fields] OR medic*[All Fields] AND revalidation[All Fields] AND English[lang]
#2	(maintenance) AND “medical licensure” Filters: English	(“maintenance”[MeSH Terms] OR “maintenance”[All Fields]) AND “medical licensure”[All Fields] AND English[lang]
#3	“maintenance of certification” Filters: English	“maintenance of certification”[All Fields] AND English[lang]
#4	“medical registration” Filters: English	“medical registration”[All Fields] AND English[lang]
#5	(maintenance) AND “professional competence” Filters: English	(“maintenance”[MeSH Terms] OR “maintenance”[All Fields]) AND “professional competence”[All Fields] AND English[lang]
#6	(doctor) AND appraisal Filters: English	(“physicians”[MeSH Terms] OR “physicians”[All Fields] OR “doctor”[All Fields]) AND appraisal[All Fields] AND English[lang]

### Selection of sources of evidence

In the initial phase of study selection, each of the four core review team members (AW, EG, IK, DB) will individually screen the titles and abstracts of all publications identified by the search strategy. Papers will be included based on the abovementioned criteria. If there is any doubt at this stage, papers will be included. Next, full-text review of selected papers will be conducted, again with reference to the inclusion criteria. Each full text will be reviewed independently by two researchers. The review team will regularly meet for face-to-face discussion of the results, of the study selection process, and to resolve any disagreements about publication inclusion. During these meetings, the team will also discuss any challenges or uncertainties about the review process and refine the search strategy if needed. A PRISMA flow diagram will be used to summarise the study selection process.

### Data charting process

Endnote X8 [21] and Microsoft® Excel [22] will be used to organise data, to manage the screening process, and to categorise and manage full-text versions of included references. A data extraction tool was developed and piloted to record information needed to answer the research questions (see Additional file 2). All members of the research team will use the tool to retrieve relevant information; the team will meet to assess whether our approach to data extraction is consistent with the research questions and purpose, and refine the data extraction tool accordingly. Cross-checking will also be undertaken to identify any inaccuracies or oversights, and any discrepancies will be discussed and resolved amongst the core review team with the involvement of the broader research team if necessary.

### Data items

In the data charting phase of the review, the following information will be collected:

- Study characteristics (e.g. author, year, title, journal, country, study aim, study design)
- Characteristics of the population studied (e.g. professional role, medical specialty)
- Key findings relating to doctors’ experiences, beliefs, and attitudes about RPC
- Key findings relating to doctors’ perceived challenges and facilitators to engagement with RPC
- Specific implications for practice or recommendations identified by study results

### Critical appraisal of individual sources of evidence

Consistent with scoping review methodology [20], no critical appraisal will be conducted for this review.

### Synthesis of results

The synthesis stage of this review will involve a descriptive summary and thematic analysis of the extracted data. The descriptive summary will include characteristics of the selected publications, types of study design, years of publication, and countries related to the publications. The thematic analysis will be similar to content analysis methods used in qualitative research methodology [23] as recommended by Levac et al. [17]. The analysis will involve identifying themes and gaps in the literature, and we may choose to use qualitative software (i.e. NVivo [24]) to facilitate this process. The final results of the scoping review will be presented through a narrative description of themes, a framework, and tables summarising pertinent information.

## Discussion

Healthcare crises and increased public and professional concerns about patient safety and quality of care have resulted in regulatory policies seeking more control, accountability, and transparency about the professional competence of doctors [7, 25, 26]. The goal of RPC, to assure patients that doctors continue to perform competently throughout their careers, is a worthy one to which all doctors would subscribe. Nonetheless, RPC in its current formats has been sharply criticised, with implications for full and meaningful engagement from doctors. Scoping reviews are useful for identifying implications for policy and practice [27].

This protocol will guide the scoping review rigorously and transparently throughout the entire process. The search strategy outlined in this protocol will ensure a comprehensive search of the literature; nevertheless, searching other databases may have identified additional relevant studies. Another limitation is the exclusion of grey literature. Following preliminary searches, it was apparent that there was a large volume of grey literature which was mostly opinion, commentary, and newspaper reports. We elected to exclude those materials so that the review would be manageable, but it is possible that some insights will have been missed as a result.

We believe that this review will be of value to those tasked with the design and implementation of RPC programmes, helping them to maximise doctors’ commitment to the process. This research is timely; internationally existing programmes are evolving, new programmes are being initiated, and many jurisdictions do not yet have programmes in place. There is an opportunity for learning across differing programmes and from the experiences of established programmes. This review will contribute to and support that learning. The findings will also be of value to researchers by pointing to areas that would benefit from further enquiry. This review is part of a larger project examining ways in which doctors’ engagement with RPC can be enhanced, and its findings will inform the other studies in this project, including a national survey of doctors in Ireland. We will disseminate the findings of the review through publications in peer-reviewed journals, conferences, and presentations.

## Additional files

Additional file 1: Prisma-ScR checklist. Checklist for reporting scoping reviews. (DOCX 106 kb)

Additional file 2: Data charting form. Form to be used for data charting process. (XLSX 9 kb)

## Data Availability

Not applicable.

## Abbreviations

CINAHL: Cumulative Index to Nursing and Allied Health Literature
CPD: Continuing professional development
PRISMA-ScR: Preferred Reporting Items for Systematic review and Meta-Analysis–Extension for Scoping Reviews
RPC: Regulation of professional competence
